# The molecular identity of fleas (Siphonaptera) carrying *Rickettsia felis, Bartonella clarridgeiae* and *Bartonella rochalimae* from dogs and cats in Northern Laos

**DOI:** 10.1016/j.heliyon.2020.e04385

**Published:** 2020-07-15

**Authors:** Nichola E.D. Calvani, Liam Bell, Abigail Carney, Carolina De La Fuente, Tori Stragliotto, Mikala Tunstall, Jan Šlapeta

**Affiliations:** Sydney School of Veterinary Science, Faculty of Science, University of Sydney, Sydney, New South Wales, 2006, Australia

**Keywords:** Microbiology, Zoology, Veterinary medicine, Infectious disease, Parasitology, Diagnostics, *Anaplasma*, cox1, gltA, omp-A, Real-time PCR, ssrA

## Abstract

Cat fleas (*Ctenocephalides felis*) are the most commonly recognised ectoparasites of domestic pets globally and are frequently implicated in the transmission of a variety of zoonotic vector-borne pathogens. The aim of the present study was to investigate the morphological and molecular identity of fleas parasitising cats and dogs in Northern Laos and screen them for a range of bacterial pathogens. Fleas (n = 120) were collected from dogs and cats and morphologically identified as *Ctenocephalides felis* (115/120)*, Ctenocephalides orientis* (4/120) and *Pulex irritans* (1/120). Molecular barcoding using the cytochrome c oxidase subunit I gene (*cox1*) was used to confirmed species identity of 21 selected fleas. The cat flea (*C. felis*) was the most dominant flea identified. *Rickettsia* and *Bartonella* spp. DNA was detected in 21/21 and 7/21 samples, respectively, via a multiplex real-time PCR targeting gltA and *ssr*A. Sequencing of the seven *Bartonella*-positive samples and ten *Rickettsia*-positive samples revealed *Bartonella clarridgeiae, Bartonella rochalimae*, *Rickettsia felis* and *Rickettsia* sp. genotype RF2125 DNA. *Anaplasma platys* DNA was detected in a single *C. felis* after 20 of the 21 DNA samples were screened using a commercial PCR panel for vector-borne pathogens. The detection of a range of bacterial pathogens in fleas from owned cats and dogs in Northern Laos provides further evidence to the importance of these ectoparasites as vectors of zoonotic diseases in the region.

## 1 Introduction

Fleas in the genus *Ctenocephalides* are agents of a variety of important zoonotic pathogens, including *Rickettsia* and *Bartonella* bacterial species (Eisen and Gage, 2012; Lawrence et al., 2019; Varagnol et al., 2009). *Rickettsia typhi* and *Rickettsia felis* cause murine typhus and flea-borne spotted fever, respectively, and *Bartonella henselae* is responsible for cat scratch disease (Eisen and Gage, 2012; Kernif et al., 2012; Varagnol et al., 2009). These vector-borne diseases are globally distributed and yet often overlooked in developing countries where access to medical care is limited. Such is the case in the Lao People's Democratic Republic where rickettsial infections have been identified in 27% of patients suffering from undifferentiated febrile illnesses, and where three molecularly-confirmed *R. felis* infections were documented between 2003-2011 (Dittrich et al., 2014; Phongmany et al., 2006).

Despite the human health implications, very little work has been conducted on the molecular identity of fleas and the pathogens they carry in Northern Laos, with previous studies relying on morphological identification alone (Kernif et al., 2012; Parola et al., 2003; Varagnol et al., 2009). The limited data available suggests that fleas in the genus *Ctenocephalides* from dogs and cats in Lao PDR are the cat flea (*Ctenocephalides felis*), the dog flea (*Ctenocephalides canis*), and the Oriental cat flea (*Ctenocephalides orientis*) (Kernif et al., 2012; Lawrence et al., 2019; Varagnol et al., 2009). The morphological differentiation of *Ctenocephalides* spp., particularly *C. orientis* and *C. canis,* requires clarification of the specimens in order to observe obscure anatomical features. Molecular barcoding of the *cox1* gene provides a straightforward alternative that has been proven to unambigiously differentiate these species (Lawrence et al., 2019).

The aim of this study was to unambiguously confirm the identity of fleas found on owned dogs and cats in Northern Laos via molecular barcoding of the *cox1* gene and to provide further evidence of their significance to human health by screening the fleas for variety of vector-borne disease pathogens with a focsus on *Bartonella* and *Rickettsia* species.

## 2 Material and methods

### 2.1 Sample collection and species identification

Fleas were opportunistically collected from two cats and seven dogs across three provinces (Xayabouli, Xieng Khouang and Luang Prabang) in Northern Laos either upon visits to the local veterinary clinic for routine vaccinations (Luang Prabang) or during livestock disease surveillance activities in the 2019 dry season. With the owner's consent the samples were collected by hand and immediately placed in Eppendorf tubes with ≥96% (v/v) ethanol for transport to the Laboratory of Veterinary Parasitology, The University of Sydney, Australia for morphological and molecular analysis.

All fleas were morphologically identified to the species level using a stereo microscope (Olympus, Australia) and published keys (Hopkins and Rothschild, 1953). Total DNA was extracted from selected individual voucher specimens (n = 21; from a maximum of three fleas per animal and at least one flea of each species) using the Isolate Genomic DNA Kit (Bioline, Australia) into a final volume of 100 μL and the exoskeleton preserved as previously described, before being clarified in KOH, dehydrated in an ethanol series and slide-mounted in Euparal for further identification to the subspecies level (Lawrence et al., 2014).

The morphological identity of each flea was confirmed molecularly via amplification of the mitochondrial encoded cytochrome c oxidase subunit I (*cox1*) as previously described (Lawrence et al., 2014). Unambiguous PCR products were sequenced at Macrogen Inc. (Seoul, Korea). Sequences were assembled, aligned with related sequences and analysed using CLC Main Workbench 6.9.1 (CLC bio, Denmark) before being deposited in GenBank (National Centre for Biotechnology Information, NCBI) under the following Accession Numbers: MT372307-MT372327.

### 2.2 Molecular detection and speciation of vector-borne pathogens including *Rickettsia* and *Bartonella* spp.

Twenty of the 21 samples were screened for vector-borne pathogens using a commercially available multiplex-tandem (MT) small animal anaemia PCR panel on the corresponding mini-plex 12 system (R910738, AusDiagnostics Pty. Ltd., Australia) as per the manufacturers’ instructions. The assay is an automated two-step nested PCR assay for the simultaneous detection of *Anaplasma platys, Babesia vogeli, Babesia gibsoni, Bartonella spp., Mycoplasma haemofelis, Mycoplasma haemocanis, Candidatus Mycoplasma* haematoparvum and *Mycoplasma haemominutum* and consists of a short (10-cycle) conventional PCR followed by a second longer SYBR-based qPCR for the detection of each of the pathogens listed (AusDiagnostics Pty. Ltd., Australia). The assay was run using 10 μL undiluted samples. Each run included controls to detect PCR inhibition (SPIKE) and sample adequacy control (ANONO) as per the recommended protocol (AusDiagnostics Pty. Ltd., Australia).

A TaqMan probe real-time PCR for *Rickettsia* and *Bartonella* species targeting the citrate synthase (*glt*A) gene and *ssr*A gene, respectively, was then used to further screen the samples for vector-borne pathogens (Diaz et al., 2012; Stenos et al., 2005). The reactions were run in duplicate in a total volume of 20 μL, with SensiFAST Probe No-ROX Kit (BioLine, Australia) and 2 μL template DNA. Real-time PCRs were run on CFX96 TouchTM Real-Time PCR Detection System (BioRad, Australia) and analysed using the corresponding CFX Maestro 1.0 software (BioRad, Australia) as previously described (Šlapeta and Šlapeta, 2016). Results were considered positive if both repeats yielded cycle threshold (Ct) values <36.00. Suspect positive results were determined when one or more repeats yielded Ct values ≥36.00 and samples were considered negative if neither repeat crossed the threshold (Ct > 40). Positive *Bartonella* results were sent to Macrogen for sequencing (Macrogen Ltd., Seoul, South Korea). Samples considered either positive or suspect positive for *Rickettsia* spp. were further identified via a pair of conventional nested PCRs targeting the outer membrane protein A (*ompA*) gene and *glt*A (Šlapeta and Šlapeta, 2016; Stenos et al., 2005). Unambiguous PCR products were sequenced at Macrogen Inc. (Seoul, Korea) before being assembled using CLC Main Workbench 6.9.1 (CLC bio, Denmark). New sequences were deposited in GenBank under the following Accession Numbers: MT394897-MT394913.

## 3 Results and discussion

A total of 120 fleas were collected from seven dogs and two cats in Northern Lao PDR in 2019, of which 21 were selected for molecular investigation (Table 1). Morphological identification revealed 115 (95.8%), four (3.3%) and one (0.8%) specimens of *Ctenocephaides felis*, *Ctenocephalides orientis* and *Pulex irritans*, respectively. Molecular barcoding at the *cox1* gene confirmed 16 (76.2%) *C. felis*, four (19.1%) *C. orientis* and one (4.8%) *P. irritans* (Table 1). All 16 *cox1* partial sequences from *C. felis* were 100% identical to each other and 100% identical to fleas from Hong Kong (KY417923), India (KX467335) and Thailand (KF684866), thus demonstrating that *C. felis* Clade 3:Tropical I (Lawrence et al., 2019) dominates dogs and cats in Lao PDR. This is in contrast to two previous studies from Lao PDR that relied on morphological keys alone to identify fleas on dogs and a cat, which suggested the dominance of *C. orientis* over *C. felis* (Varagnol et al., 2009; Kernif et al., 2012). No *C. canis* were identified in the current study, confirming our previous finding that *C. canis* is rare or absent from South East Asia (Lawence et al., 2019). We recommend the routine confirmation of morphologically identified *C. canis/C. orientis* using a *cox1* DNA approach, because it is possible that past and current records of *C. canis* are misidentified *C. orientis* (Colella et al., 2020; Lawrence et al., 2014, 2019).

**Table 1:** Location, host species, flea species, number and sex of fleas (M/F) collected from owned cats and dogs in Northern Laos.

Identifier	Host	Locality in Lao PDR	*C. felis*	*C. orientis*	*P. irritans*	*Samples for DNA analysis*
P61/19-A	*Canis familiaris*	B. Nam Tuan, Xayabouli	1	2	0	P61/19-A1 (F), A2 (F), A3 (M)
P61/19-B	*Felis catus*	SK Clinic, Luang Prabang	1	0	0	P61/19-B1 (F)
P61/19-C	*Canis familiaris*	B. Konekham, Luang Prabang	22	0	0	P61/19-C1 (M), C2 (M), C3 (F)
P61/19-D	*Canis familiaris*	B. Soynafar, Xieng Khouang	1	1	1	P61/19-D1 (M), D2 (F), D3 (M)
P61/19-E	*Felis catus*	B. Nong, Xieng Khouang	6	0	0	P61/19-E1 (F), E2 (F), E3 (M)
P61/19-F	*Canis familiaris*	SK Clinic, Luang Prabang	1	0	0	P61/19-F1 (F)
P61/19-G	*Canis familiaris*	SK Clinic, Luang Prabang	1	0	0	P61/19-G1 (F)
P61/19-H	*Canis familiaris*	B. Soynafar, Xieng Khouang	4	1	0	P61/19-H1 (F), H2 (F), H3 (F)
P61/19-I	*Canis familiaris*	DLF Lab, Luang Prabang	78	0	0	P61/19-I1 (F), I2 (F), I3 (M)

The *cox1* partial sequences from four *C. orientis* were 100% identical to each other and 100% identical to *cox1* of *C. orientis* from China (MG586605), Thailand (MG586338) and Bhutan (MG586534). The *cox1* sequences were identical to those previously identified in Lao PDR, and according to standardized *cox1* haplotype names (Lawrence et al., 2019), the *C. felis* and *C. orientis* in the current study correspond to h2 (MG586362) and h4 (MG586363), respectively. The *cox1* partial sequence from the single *Pulex irritans* was 100% identical to *cox1* from *P. irritans* in New Zealand (KY048351), Iran (MF380391) and Spain (LT797470). This study, as well as the previous studies (Kernif et al., 2012; Varagnol et al., 2009), was based on a small number of examined animals and more sampling of dogs and cats will be required to draw further conclusions on the apparent lack of within-species genetic diversity. For example, extensive sampling across dogs and cats in Australia demonstrated the presence of three *C. felis* haplogroups with distinct ecological preferences (Crkvencic and Šlapeta, 2019; Šlapeta et al., 2011).

The MT-PCR panel used to simultaneously detect eight different anaemia-causing pathogens of dogs and cats revealed the presence of *A. platys* DNA in a single (5%, 1/20) flea, *C. felis* (P61/19-C2), collected from a dog in Luang Prabang Province. Although this is an interestesting finding, further investigation is needed to better understand the role of fleas in the epidemiology of *A. platys*, which is currently poorly understood (Carrade et al., 2009). The panel detected *Bartonella* spp. DNA in half of the fleas tested (10/20), and all three *C. felis* samples collected from one of the two cats sampled tested positive for *Bartonella* DNA (P61/19-E1 to E3) (Table S1). All of the fleas tested (n = 20) were negative for the remaining targets. While there is a lack of information available regarding the diversity of vector-borne pathogens in fleas and their companion animal hosts in Lao PDR, a study of canine vector-borne pathogens in 101 semi-domesticated dogs in Cambodia revealed a high prevalence of *B. vogeli* (32.7%), *M. haemocanis* (9.9%) and *Candidatus Mycoplasma* haematoparvum (2.9%), none of which were detected in the current study (Inpankaew et al., 2016). These differences might be attributed to the limited sample size of the current study and provides further evidence towards increased surveillance of vector-borne diseases within ectoparasites and their mammalian hosts in Lao PDR. Further work within Southeast Asia is needed to refine our understanding of vector borne disease and arthropods parasitising dogs and cats (Colella et al., 2020).

We then screened all 21 fleas for the presence of *Bartonella* and *Rickettsia* spp. DNA using a multiplexed real-time PCR assay for the simultaneous detection of both pathogens (Table 2). Seven (7/21) samples were considered at least suspect positive (Ct values <40.00) for both targets, one of which was from the single *Pulex irritans* sample and six (6/20) *Ctenocephalides* spp. DNA samples were positive for *ssr*A *Bartonella* spp. DNA. The *ssr*A DNA sequences from *C. felis* (n = 6) were 100% identical to each other and to *ssr*A from a *Bartonella clarridgeiae* strain Houston-2 (JN982716). This finding is in alignment with earlier work identifying *Bartonella* spp. in Lao PDR, although is higher than was previously detected (5/54 of *Ctenocephalides* spp.) further south on the Thai-Myanmar border (Angelakis et al., 2009; Parola et al., 2003; Varagnol et al., 2009). All *B*. *clarridgeiae* positive samples were from *C. felis*, while no *Bartonella* spp. DNA was detected in any of the four *C. orientis* samples. The *Bartonella* spp. DNA sequence amplified from the total DNA of the single *P. irritans* sample showed 100% similarity to *ssr*A from *Bartonella rochalimae* type strain BMGH (JN029797), which is the first time this species has been detected in Laos.

**Table 2:** Summary of *Rickettsia* and *Bartonella* diagnostic results from fleas collected off owned dogs and cats in Northern Laos. Pathogen species are indicated by the asterix annotations ∗*Rickettsia* sp. Genotype RF2125, ∗∗*Rickettsia felis*, ∗∗∗*Bartonella clarridgeiae* and ∗∗∗∗*Bartonella rochalimae*.

Identifier	Host	Flea species	Number of fleas tested	*Number of samples PCR positive*	
				*Rickettsia*	*Bartonella*
P61/19-A	*Canis familaris*	*Ctenocephalides felis*	1	1/1	0/1
		*Ctenocephalides orientis*	2	2/2∗	0/2
P61/19-B	*Felis catus*	*Ctenocephalides felis*	1	1/1∗∗	0/1
P61/19-C	*Canis familaris*	*Ctenocephalides felis*	3	3/3∗∗	0/3
P61/19-D	*Canis familaris*	*Ctenocephalides felis*	1	1/1	1/1∗∗∗
		*Ctenocephalides orientis*	1	1/1	0/1
		*Pulex irritans*	1	1/1∗∗	1/1∗∗∗∗
P61/19-E	*Felis catus*	*Ctenocephalides felis*	3	3/3∗∗	2/3∗∗∗
P61/19-F	*Canis familaris*	*Ctenocephalides felis*	1	1/1∗∗	1/1∗∗∗
P61/19-G	*Canis familaris*	*Ctenocephalides felis*	1	1/1∗∗	1/1∗∗∗
P61/19-H	*Canis familaris*	*Ctenocephalides felis*	2	2/2	0/2
		*Ctenocephalides orientis*	1	1/1∗∗	0/1
P61/19-I	*Canis familaris*	*Ctenocephalides felis*	3	3/3∗∗	1/3∗∗∗

All (20/20) *Ctenocephalides* spp. DNA samples were *gltA Rickettsia* spp. qPCR positive and negative controls remained negative, ruling out potential cross-contamination. DNA from nine fleas was further tested using a nested PCR targeting *gltA* and *ompA* genes for additional *Rickettsia* spp. identification. DNA sequencing of the PCR products confirmed that the DNA from *C. orientis* (P61/19-A1) 100% matched *gltA* DNA from *Rickettsia* sp. genotype RF2125 (KX431979), which was initially detected along the Thai-Myanmar border (Parola et al., 2003). At *ompA* the P61/19-A1 sequence was 100% identical to *Rickettsia* sp. genotype RF2125 (KP256359) from *C. orientis* from India. The *gltA* and *ompA* from the remaining fleas (seven *C. felis,* one *C. orientis* and one *P. irritans*) matched 100% with sequences from the reference genome of *R. felis* (CP000053). Such a high prevalence of *R. felis* in the current study is in agreement with other findings from Lao PDR and adds further evidence to the importance of this pathogen as a cause of undifferentiated febrile illnesses in human patients (Dittrich et al., 2014; Kernif et al., 2012; Phongmany et al., 2006; Varagnol et al., 2009).

This study presents the first application of molecular techniques for the identification of fleas and the pathogens they carry in Lao PDR and suggests that both dogs and cats are important reservoirs of known zoonotic pathogens, specifically *Bartonella* and *Rickettsia* spp. These findings, and the abundance of *R. felis* in particular*,* have implications for the management of malaria and dengue-negative febrile patients and support the use doxycycline when more extensive diagnostic services and medical care is unavailable (Dittrich et al., 2014; Phongmany et al., 2006; Syhavong et al., 2010).

## Declarations

### Author contribution statement

Nichola E.D. Calvani: Conceived and designed the experiments; Performed the experiments; Analyzed and interpreted the data; Wrote the paper.

Liam Bell, Abigail Carney, Carolina De La Fuente, Tori Stragliotto, Mikala Tunstall: Performed the experiments; Analyzed and interpreted the data.

Jan Šlapeta: Conceived and designed the experiments; Analyzed and interpreted the data; Contributed reagents, materials, analysis tools or data; Wrote the paper.

### Funding statement

This research did not receive any specific grant from funding agencies in the public, commercial, or not-for-profit sectors.

### Competing interest statement

The authors declare no conflict of interest.

### Additional information

No additional information is available for this paper.
